# Repurposing Melt Degradation for the Evaluation of Mixed Amorphous-Crystalline Blends

**DOI:** 10.1208/s12249-021-01971-6

**Published:** 2021-03-14

**Authors:** Sumayah Abdul-jabbar, Daniel W. Wong, Gary P. Martin, Brendon Woodhead, Paul G. Royall

**Affiliations:** 1 School of Cancer and Pharmaceutical Sciences, Institute of Pharmaceutical Science, King’s College London, Franklin-Wilkins Building, 150 Stamford Street, London, SE1 9NH UK; 2 SciCore Industries, 20 Thomas Hamer Place, Auckland, 0632 New Zealand

**Keywords:** salbutamol sulphate, amorphous content, differential scanning calorimetry, melt degradation, amorphous-crystalline blends

## Abstract

Medicine regulators require the melting points for crystalline drugs, as they are a test for chemical and physical quality. Many drugs, especially salt-forms, suffer concomitant degradation during melting; thus, it would be useful to know if the endotherm associated with melt degradation may be used for characterising the crystallinity of a powder blend. Therefore, the aim of this study was to investigate whether melt-degradation transitions can detect amorphous content in a blend of crystalline and amorphous salbutamol sulphate. Salbutamol sulphate was rendered amorphous by freeze and spray-drying and blended with crystalline drug, forming standards with a range of amorphous content. Crystalline salbutamol sulphate was observed to have a melt-degradation onset of 198.2±0.2°C, while anhydrous amorphous salbutamol sulphate prepared by either method showed similar glass transition temperatures of 119.4±0.7°C combined. Without the energy barrier provided by the ordered crystal lattice, the degradation endotherm for amorphous salbutamol sulphate occurred 50°C below the melting point, with an onset of 143.6±0.2°C. The enthalpies for this degradation transition showed no significant difference between freeze- and spray-dried samples (*p*>0.05). Distinct from convention, partial integration of the crystalline melt-degradation endotherm was applied to the region 193–221°C which had no contribution from the degradation of amorphous salbutamol sulphate. The linear correlation of these partial areas with amorphous content, *R*^2^=0.994, yielded limits of detection and quantification of 0.13% and 0.44% respectively, independent of drying technique. Melt-degradation transitions may be re-purposed for the measurement of amorphous content in powder blends, and they have potential for evaluating disorder more generally.

## INTRODUCTION

Numerous analytical techniques have been reported for the detection and evaluation of the amorphous phase, but issues of suitability pose significant challenges (1, 2). Powder X-ray diffraction (PXRD) is recognised as the industry ‘gold-standard’. However, it can be difficult to quantify amorphous contents below 5% (3). Since the amorphous form always differs from the crystalline state in terms of physical stability, calorimetric methods are frequently used to evaluate crystallinity and amorphous content by recording glass transitions and the extent of recrystallisation and melting (4).

The use of differential scanning calorimetry (DSC) enables amorphous content to be detected if the presence of a glass transition is observed within the heating cycle (5), by measuring the heat capacity change associated with this transition (2, 6). In addition, the amorphous material may recrystallise thereby producing an exothermic crystallisation peak (7) which can also be measured for amorphous content detection (5). When low levels of amorphous content are being investigated, in an otherwise chemically pure system, if the law of mass balance is considered, there should be a concomitant greater fraction of the material which is crystalline. In the case of polymers which contain both amorphous and crystalline regions, the size of any melting peak detected is frequently used to determine the fraction of crystallinity present within the material (8). Once the fraction of crystallinity is measured, it is straightforward to determine the amorphous content as both fractions, if no other phases are present, must sum to one.

Using the melting endotherms of drug substances is not frequently considered as an approach for detecting amorphous content in pharmaceutical development. This is peculiar as very often the largest signal observed in a differential scanning calorimeter is that of the melting peak, which has a high signal-to-noise ratio and thus the potential for low limits of detection. Occasionally, the enthalpy of fusion has been incorporated with the heat flow observed for recrystallisation to determine amorphous content (9) but using the reduction in the size of the melting peak alone, when process-induced disorder is suspected, is seldom considered (10). One barrier that is preventing the uptake of this approach is the potential for degradation upon melting (11).

Thus, in the research reported here, the melting of a drug substance that has a known and well-characterised melt-degradation transition, salbutamol sulphate (SS), was investigated. SS has been shown to be thermally stable up to 180°C but upon melting chemical decomposition is known to occur (12).

An essential first step in the evaluation of melt-degradation transitions for amorphous content detection is the study of powder blends. Crystalline and amorphous powder blends are frequently used in the development of protocols for amorphous content detection and thus they are important tools for characterising process-induced disorder and product stability (13).

Therefore, the aim of this study was to investigate whether heat-induced melt-degradation may be used to detect amorphous content in blends of crystalline and amorphous SS and to evaluate how two different production methods for the amorphous form of the drug may influence the thermal transitions observed.

## MATERIALS AND METHODS

### Materials

Crystalline salbutamol sulphate (SS) (> 99%) (GlaxoSmithKline) and HPLC gradient water (Fischer Scientific) were used as received.

### Methods

#### Production of Amorphous Standards of SS

A 10% w/v solution of SS was used for both freeze and spray-drying. Samples were loaded into vials with pierced caps and stored at −75°C for 30 min. Freeze-drying was then conducted overnight using Ehrist ALPHA I-5 Freeze-dryer.

SS solutions were fed into the spray-drier Büchi Mini Spray-Drier B-191. The operating parameters were as follows: a feed rate of 20 mL/min, air flow of 67 kg/h (14), an inlet temperature of 160±3°C, and an outlet temperature of 82±1°C.

#### Preparation of Amorphous-Crystalline Powder Blends

Crystalline unprocessed SS was mixed with amorphous SS to produce powder blends with amorphous content ranging from 1 to 100% w/w. Blending was affected by triturating small amounts of crystalline and amorphous drug together in glass vials and a homogenous powder mix was produced by applying the vials to a Whirlimixer for 30 s. The above steps were then repeated until the required final ratio was achieved.

#### Scanning Electron Microscope

Quanta 200F field emission SEM operated in high vacuum mode (5 kV) was used. The powder-covered stubs were then sputter coated with gold using a Palaron E5100 sputter coater.

#### Differential Scanning Calorimetry

Samples were analysed using TA instruments DSC 2920. Experiments were run using sealed aluminium DSC pans containing approximately 1–4 mg of powder with a ‘pin-holed’ lid. Samples were heated over a temperature range of 28–230°C at a heating rate of 10°C/min. The purge gas used was nitrogen at a flow rate of 55 mL min^−1^. The recorded heat flow was normalised with respect to the sample loading mass and also offset in their plotting for clarity purposes.

## RESULTS

### Scanning Electron Microscopy

**Fig. 1:**
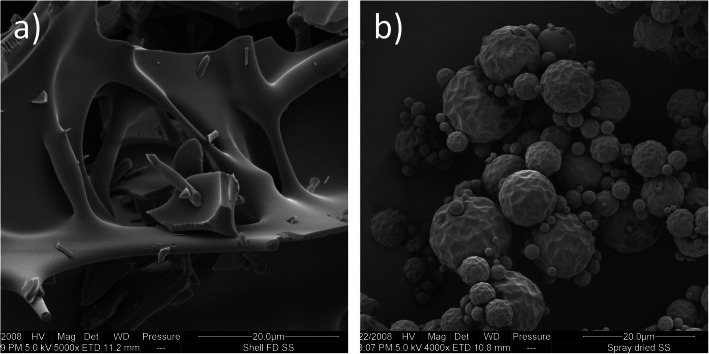
SEM micrographs of **a **freeze-dried SS and **b **spray-dried SS

The two procedures for preparing amorphous SS resulted in the production of particles with obvious differences in shape (Fig. 1). Well-defined spheres of spray-dried SS were produced using spray-drying as opposed to the contiguous porous structure of the fragments typically generated by freeze-drying.

**Fig. 2:**
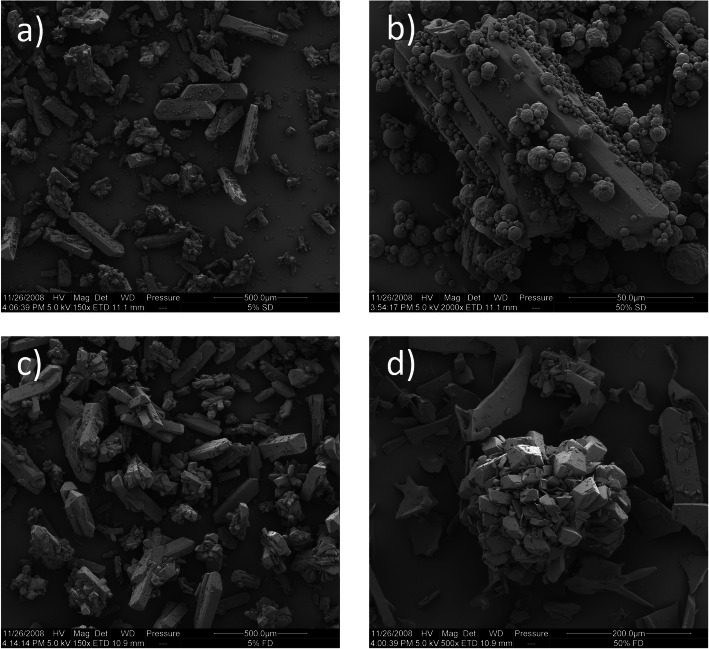
SEM micrographs of **a **×150 magnification of 5% spray-dried, **b **×2000 magnification of 50% spray-dried, **c **×150 magnification of 5% freeze-dried, and **d **×500 magnification of 50% freeze-dried powdered samples of SS mixed with the crystalline material

The relative efficiency of the mixing process is apparent from the SEM of the freeze- and spray-dried particles mixed with different amounts of crystalline material (Fig. 2), where an apparent random mixture of the different morphological forms was manifest.

For the 5% blends, the spray-dried smaller particles appear to be evenly spread on the surfaces of the larger crystals of SS, but when the blend is 50%, both the spray-dried spheres and freeze-dried fragments adhered to the columnar crystals together to form larger aggregates.

### Differential Scanning Calorimetry and the Quantification of Amorphous Content

**Fig. 3:**
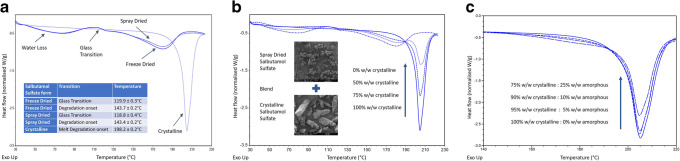
**a** Overlay of thermograms comprising samples of crystalline, freeze-, and spray-dried SS. **b** Overlay of thermograms comprising amorphous (0%), crystalline (100%), and 50 and 75% blends of crystalline and amorphous SS. **c** Overlay of thermograms comprising amorphous (0%) and 5, 10, and 25% blends of crystalline and amorphous SS

The endothermic transitions are presented as troughs in all the DSC thermograms (Fig. 3). The thermogram of the crystalline material (Fig. 3a) indicates that little change is detectable in the baseline between 30 and 175°C. Melting or degradation of salbutamol sulphate is depicted by the endothermic peak at 198.2 ±0.2°C. Two endothermic peaks can be seen for both the freeze- and spray-dried salbutamol salt. The first peak between 25 and 100°C indicates the loss of residual water from the material, whereas the second peak at 143.6±0.2°C can be attributed to melting or degradation. A step change in baseline, which is typical of a glass transition, was seen at 119.4±0.7°C. DSC can measure the reduction in melt degradation of the endothermic peak due to crystalline content as the amorphous content is increased, Fig. 3b showing the effect of adding higher concentrations and Fig. 3c showing the influence of lower concentrations. It should be noted also that the step change of the glass transition becomes smaller as the % amorphous content decreases.

The linear regression analysis showed a correlation between the mean integrated peak and % amorphous content with *R*^2^ values of 0.999 and 0.994, respectively for freeze- and spray-dried SS (no significant difference (paired *t* test, *p*> 0.05)).

**Fig. 4:**
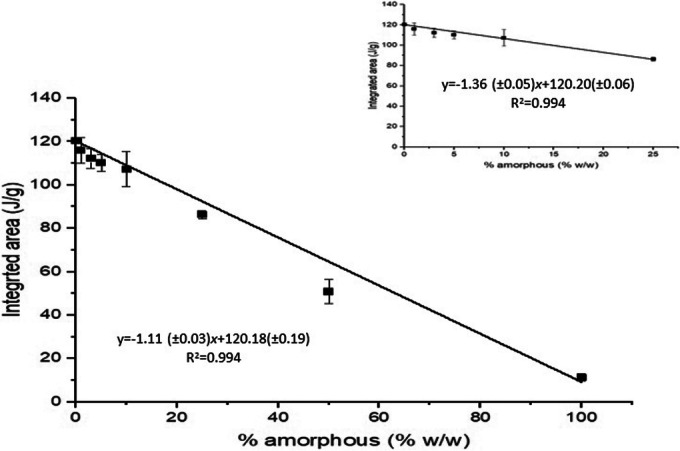
Linear fit of mean DSC integrated peak area from 193 to 221°C against % amorphous content (*n*=6). The inset shows the samples with a 0–25% amorphous content plotted on a larger scale

The mean integrated area for both freeze- and spray-dried was plotted against % amorphous content and regression analysis was conducted (Fig. 4) showing a linear relationship. Limits of detection (LOD) and quantification (LOQ) of 0.13% and 0.44%, respectively, of amorphous content were calculated.

## DISCUSSION

In this study, a method was developed based on employing DSC to determine the thermographs of powders comprising mixtures of amorphous and crystalline material. These results complement those reported previously where calorimetry results were compared to those obtained from a variety of other methods, designed to determine the amorphous content of powdered salbutamol sulphate (2).

SEM showed that the freeze- and spray-drying methods produced materials possessing distinctly different morphologies (Fig. 1). The generally columnar shape of the crystal structure of the salt was lost upon spray- and freeze-drying (2). The distribution of amorphous material on both the surface of and between the SS crystals can be seen clearly, indicating adequate mixing (Fig. 2).

The thermograph of the unprocessed crystalline material displayed a single endothermic peak temperature of 198.2 ± 0.2 °C (Fig. 3a) which is comparable to the value obtained in literature (15, 16) indicating that it was pure. The endothermic events at approximately 200°C have been interpreted differently previously involving melting of the crystal followed by degradation or alternatively to various stages of drug degradation. The amorphous SS showed no evidence of recrystallisation occurring in any of the samples examined.

In this study, the contents of the pans were not analysed during and after heating and thus it was assumed that the endotherms post-Tg correspond to both melting and degradation of SS. Previous studies conclude that the melting endotherm of SS also includes the degradation of the drug via the elimination of the tertiary butylamine group (17). This hypothesis agrees well with the observation that the heated material was dark yellow in colour, in contrast to its initial white colour. Therefore, it is likely that the second endotherm present in the thermographs of the amorphous samples was related to drug degradation of this type. In another study, which utilised thermal analysis coupled to other analytical techniques, it was proposed that SS undergoes a multi-step degradation mechanism in which the molecule is dehydrated, leading to the initial loss of water. As heating continues, this is followed by the break-up of the secondary amine group leading to the release of ammonia and then lastly to the liberation of sulphur dioxide (18). Such a pathway of degradation was supported by a preliminary study conducted here whereby TG-MS data confirmed that when salbutamol melted it underwent degradation evolving several volatile species including water, sulphur dioxide, and sulphur trioxide (data not shown).

Derivative thermogravimetric data have indicated that salbutamol sulphate is thermally stable up to 180°C. However, as the temperature was raised between 180 and 600°C, successive mass losses occurred, i.e. at 204°C thermal decomposition began (18% mass loss), then thermal decomposition of the sample occurred at 299°C with carbonisation of the compound (4% mass loss), and finally at 580°C, elimination of carbonisation products resulted (11% mass loss) (16). Thermogravimetic analysis, conducted on the crystalline salbutamol sulphate as part of the current study, supported this previously reported data, with a 12.5% mass loss being found at 200°C (data not shown).

Even though the topography and shape of the amorphous SS prepared by freeze- and spray-drying were extremely different, the two preparations did appear to have similar thermal properties. Therefore, it could be concluded that the preparation method of the amorphous material had no influence on the melt degradation endotherm.

The work reported here shows an excellent correlation between the partial integral area, between 193 and 221°C, the region of the endothermic peak corresponding to melt/degradation of the crystalline fraction and the total amorphous content of the blends. It should be noted that amorphous materials degrade at lower temperatures, due to the lowering of the thermodynamic barrier towards degradation in comparison to the corresponding crystalline materials. This is because the heat input, (enthalpy of fusion) that is required to disturb the ordered crystal lattice and cause breakage of the noncovalent bonds which hold the molecules in position, is not needed. Such a mechanism is evident for amorphous SS, with a degradation onset of 143.6±0.2°C occurring some 50°C below the melting point of the crystalline form of the drug. The overall area of the endothermic peak associated with melt degradation decreased as the amount of amorphous content increased (Fig. 3b and 3c).

A linear regression analysis of the combined DSC results derived from samples processed by freeze- and spray-drying showed a linear correlation between the mean partial integrated peak areas and % amorphous content, with *R*^2^ value of 0.994 (Fig. 4). Thus, in the samples investigated in the present study, there was weight additivity with respect to the partial integral area associated with melt degradation of the crystalline fraction. The linear relationship implies that the amorphous fractions of the blends degrade at a lower temperature without affecting the melt degradation of the crystalline part, as the phase separation is maintained within the heating cycle.

## CONCLUSION

Heat-induced melt-degradation has been used successfully to detect amorphous content in crystalline-amorphous powder blends prepared by both freeze- and spray-drying. The preparative route for the amorphous drug used in the blends did not appear to influence thermal stability or the heat flow observed in the calorimeter. In a novel approach, partial integration of the melt degradation endotherm was applied to the region of the heat flow signal which had no contribution from amorphous degradation. In the case of salbutamol sulphate, this was between 193 and 221°C. Thus, melt-degradation transitions may be used to measure amorphous content in powder blends, and they have potential for evaluating disorder more generally. More studies are required to investigate whether this concept can be extended to characterise a range of other materials of pharmaceutical interest.
